# On Cancerous Affections of the Skin

**Published:** 1886-12

**Authors:** 


*On Cancerous Affections of the Skin.* By GEORGE THIN, M.D. Pp. 87. London: J. & A. CHURCHILL. 1886.

Dr. Thin's original researches in the histology of cancerous affections of the skin are so valuable and so generally known, that this monograph, embodying much of his original work, and bringing the whole subject under review in limited compass, will be gladly welcomed.

The original histological part of the work is, of course, the most valuable. But the sections devoted to description, diagnosis, and treatment, are clear, sensible, sufficiently full, and quite up to date. The book will well repay perusal, and is worthy of a permanent place on the bookshelves.

